# A practical measure of health facility efficiency: an innovation in the application of routine health information to determine health worker productivity in Ethiopia

**DOI:** 10.1186/s12960-021-00636-6

**Published:** 2021-08-05

**Authors:** Md Zabir Hasan, Girmaye D. Dinsa, Peter Berman

**Affiliations:** 1 School of Population and Public Health, University of British Columbia, 2206 E Mall, Vancouver, BC V6T 1Z3 Canada; 2 Department of Global Health and Population, Harvard T. H. Chan School of Public Health, Boston, MA USA; 3 Department of Public Health and Health Policy, College of Health Sciences, Haramaya University, Harar, Ethiopia

**Keywords:** Efficiency, Technical efficiency, Productivity, Health centers, Primary healthcare, Health information management system, Health personnel, Factor analysis, Ethiopia, Low- and middle-income countries

## Abstract

**Background:**

A simple indicator of technical efficiency, such as productivity of health workers, measured using routine health facility data, can be a practical approach that can inform initiatives to improve efficiency in low- and middle-income countries. This paper presents a proof of concept of using routine information from primary healthcare (PHC) facilities to measure health workers’ productivity and its application in three regions of Ethiopia.

**Methods:**

In four steps, we constructed a productivity measure of the health workforce of Health Centers (HCs) and demonstrated its practical application: (1) developing an analytical dataset using secondary data from health management information systems (HMIS) and human resource information system (HRIS); (2) principal component analysis and factor analysis to estimate a summary measure of output from five indicators (annual service volume of outpatient visits, family planning, first antenatal care visits, facility-based deliveries by skilled birth attendants, and children [< 1 year] with three pentavalent vaccines); (3) calculating a productivity score by combining the summary measure of outputs and the total number of health workers (input), and (4) implementing regression models to identify the determinant of productivity and ranking HCs based on their adjusted productivity score.

**Results:**

We developed an analytical dataset of 1128 HCs; however, significant missing values and outliers were reported in the data. The principal component and factor scores developed from the five output measures were highly consistent (correlation coefficient = 0.98). We considered the factor score as the summary measure of outputs for estimating productivity. A very weak association was observed between the summary measure of output and the total number of staff. The result also highlighted a large variability in productivity across similar health facilities in Ethiopia, represented by the significant dispersion in summary measure of output occurring at similar levels of the health workers.

**Conclusions:**

We successfully demonstrated the analytical steps to estimate health worker productivity and its practical application using HMIS and HRIS. The methodology presented in this study can be readily applied in low- and middle-income countries using widely available data—such as DHIS2—that will allow further explorations to understand the causes of technical inefficiencies in the health system.

**Supplementary Information:**

The online version contains supplementary material available at 10.1186/s12960-021-00636-6.

## Background

Advancing towards universal health coverage requires high-quality, equitable, and affordable health services, with an emphasis on the primary healthcare (PHC) system [1]. Improving the availability, accessibility, and coverage of PHC requires an increase in fiscal space for health-budgetary allowance to allocate additional resources for health without compromising the other sectors’ financial sustainability [2]. Government health expenditure can be increased by economic growth, reprioritizing budgetary allocation, and generating additional revenue (such as earmarked taxation) [3]. However, these strategies depend on the broader macroeconomic policy, political environment, and cross-sectoral priorities. Historically, foreign aid and philanthropic contributions have supported the low- and middle-income countries (LMICs) to expand their fiscal space, but we observe a declining trend of health sector-specific development assistance [4]. Improving the efficiency of healthcare delivery in making better use of scarce resources is an important strategy to meet today’s needs [5].

In its simplest form, the efficiency of health service provision is the result of how well resources (inputs) are used to produce outputs (e.g., service provided) and outcomes (e.g., gained health benefits, or return of investment) [6]. A health system can gain efficiency through some combination of improving its allocative efficiency (AE) and technical efficiency (TE). AE results when the health systems inputs are organized to optimize outcomes. Impact evaluation of healthcare interventions puts AE at the center of their interest. Indicators such as “cost per quality-adjusted life-years” are measured using cost-effectiveness analysis to understand the AE of interventions [6]. In contrast, TE results when health service outputs are produced with a minimal level of inputs or at least cost. While AE focuses on the strategic choice of interventions to maximize outcomes, TE emphasizes the operational aspect of the health systems by assessing the variability of inputs required in relation to the outputs [6]. At the health facility level, measures such as bed occupancy or staff productivity highlight TE [7].

Over the last decades, significant improvement in the AE of PHC services was achieved by organizing service delivery to emphasize better population health gain in LMICs [8, 9]. In relation to TE, in 2010, the World Health Organization estimated that 20–40% of health resources was wasted due to inefficiency associated with inappropriate use of medicine, procedures and logistics, low quality of care, and suboptimal health workers productivity [10]. With resources increasing under stress, TE of service provision is becoming a critical strategy to assure sufficient resources for health in the LMICs.

### Challenges in measuring technical efficiency of primary healthcare provision in low- and middle-income countries

**Fig. 1:**
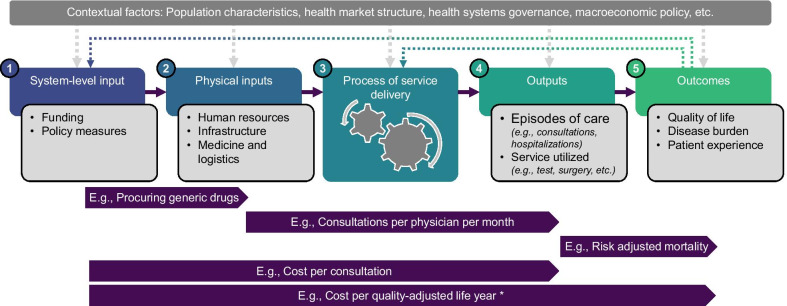
A simplified diagram presenting the health service provision process and indicators of technical efficiency. *Note: The framework is * *adapted from Cylus *et al., 2017; * Cost per quality-adjusted life year is an allocative efficiency indicator

Healthcare provision in multi-function health facilities can be characterized as a “multiple-input multiple-output” production process. That is, different types of inputs (e.g., human resources, equipment, supplies) are combined to produce many outputs (e.g., treatments of acute illness, maternity care, immunizations). In this paper, we focus specifically on processes related to the TE of output production in relation to the level of the health workforce (Fig. 1: Box 2–4).

Various methods can be used to trace the TE of this process, such as cost assessment or multivariate production modeling [11]. The results of these analyses could be used to develop payment methods to incentivize more efficient behavior, such as diagnostic-related groups (DRGs) for hospital payment systems [12]. In contrast, analytical methods such as data envelopment analysis (DEA) and stochastic frontier analysis (SFA) can deal with the “multiple-input multiple-output” problem [13]. DEA and SFA benchmark facilities against those lying close to the production possibility frontier, representing TE [14]. Though both methods are statistically sound when applied to good quality data, they are most commonly applied by academics and scarcely used for regulatory purposes by health systems managers and policymakers due to their statistical complexity [6]. Furthermore, from the perspective of policymakers or health system managers, aiming to improve the efficiency of all health facilities to the highest observed level—the production possibility frontier identified by DEA and SFA—may not be the most practical approach to achieve better results. A focus on those facilities with below average performance first may be more feasible given the available resources.

Moreover, most LMICs lack individual patient-based records, which would enable detailed costing of services. Empirical costing studies implemented in the PHC setting use a variety of tools to collect data for estimating the cost of services [15]. However, data collection is often expensive and not feasible to scale up with rapid feedback for health system management [16]. PHC facilities in many LMICs maintain regular reporting of health service inputs and outputs through their health management information systems (HMIS) and facility-based registers. Practical methods to analyze these data to generate feedback on TE could provide the basis for initiatives to improve efficiency.

This paper presents an example of how regularly reported data elements such as, the number of health workers and the volume of service they provide—can give us critical insight into their productivity, which is a measure of TE. We are presenting a proof of concept of using routine information from PHC facilities to measure health workers’ productivity and its application from three regions of Ethiopia.

## Methods

### Study setting and data source

This research aims to examine the relationship between the size and composition of the health workforce (input) and the volume of service utilization (output) of Health Centers (HCs) from three regions of Ethiopia: Addis Ababa, Oromiya, and Southern Nations, Nationalities and People’s Region (SNNPR). We used secondary data of Ethiopian Fiscal Year 2009 (Gregorian Calendar 2016) from three sources: the HMIS for outputs [17], Human Resource Information System (HRIS) for inputs and facility attributes [18, 19], and subnational-level (woreda or district) population projections of Ethiopia from the US Census Bureau’s International Data Base [20].

HCs in Ethiopia provide both preventive and curative services—including family planning, perinatal care, facility-based delivery, vaccination services, and outpatient consultations. Some HCs also provide limited inpatient service with five beds. To provide the wide range of PHC services mentioned above, on average, 20 healthcare providers and allied staff are posted in each HCs [21]. The staffing includes emergency surgical officers, health officers (HOs), nurses, midwives, pharmacy professionals, laboratory technicians, and administrative staff [22]. While there are few HCs where doctors are posted, in most HCs, clinical service is provided by non-physician health workers such as HOs and emergency surgical officers [23].

### Measurements

#### Output measures

We selected five indicators representing the annual volume of curative and reproductive, maternal, and child health services provided by the HCs—number of outpatient visits (OPD), number of acceptors of modern family planning methods (FP), total first antenatal care visits (ANC1), annual number of facility-based deliveries by skilled birth attendants (SBA), and number of children with three pentavalent vaccines received within their first year (PENTA3). HCs reported monthly volume of these services through the HMIS system in 2016 [21]. We acquired the annual service utilization of the five indicators from 2163 HCs, along with their name and geographical locations from the HMIS repository.

#### Input measures

The HRIS reports the number of all types of healthcare workers posted in each HC at the beginning and the end of the fiscal year [18]. We identified 2005 HCs from the three regions and retrieved the health workforce information at the beginning of the fiscal year 2016, along with the name and geographical locations of the HCs. We categorized all healthcare workers of the HCs into three groups—clinical, para-clinical, and administrative staff (see Additional File 1 for more details on healthcare worker categorization). To adjust the variability of the skills of health service provision by the different clinical staff, we constructed the number of HO equivalent clinical staff for each HCs.

HOs are assigned as the primary clinical service providers in the HCs in Ethiopia after receiving 4 years of clinical pre-service training [22]. On the contrary, medical doctors and emergency surgical officers receive 6 years, nurses receive 4 years, and midwives received 3 years of clinical pre-service training [23, 24]. Considering each HO as one clinical staff, we calculated the sum of weighted-clinical staff by assigning a weight of 1.5, 1.5, 1.0, and 0.75 for each doctor, emergency surgical officer, nurse, and midwife posted in the HCs, accordingly. The sum of weighted-clinical, para-clinical, and admin staff represents the total workforce of an HC.

#### Contextual covariates

Many contextual factors can also confound the estimation of productivity of health workers [25]. These factors can be either facility’s intrinsic characteristics [26]—for example, infrastructure, provider-mix, financing, management, etc.—or extrinsic factors such as geography, demography, and the healthcare market structure [27, 28]. As intrinsic factors, we included the number of beds of the HCs as a proxy for facility size and the provider-mix of clinical, para-clinical, and admin staff. As extrinsic contextual covariates, we included the geographical location of the HCs and the woreda population where the HC is situated, estimated by the US Census Bureau [20].

### Analytical approach

To develop the productivity measure of the health workforce, we followed these analytical steps: (1) development of the analytical dataset; (2) estimating a summary measure of the five outputs, and exploring its distribution; (3) constructing a productivity ratio by using the summary measure and total staff, and exploring its characteristics, and (4) providing two examples of practical applications of the productivity ratio that could be a part of routine health service monitoring and provide the basis for interventions to improve TE.

We exported the input and output measures of the HCs, and the woreda-level population estimates from Microsoft Excel spreadsheets to Stata 15.1 [29] for data management. We performed data cleaning by checking the frequency and missingness and found that some HCs reported a high volume of utilization and staff numbers. We identified outliers from the input and output measures using the interquartile range (IQR) method [30]. After performing listwise deletion of any missing and outliers, 1582 HCs with all five output measures and 1,483 HCs with the input information remained. Combining the output and input measures by matching the name of the facility and location (woreda and zone), we developed a unified dataset of 1128 HCs and merged the woreda-level population estimates with the dataset.

### Developing a summary measure of output

**Fig. 2:**
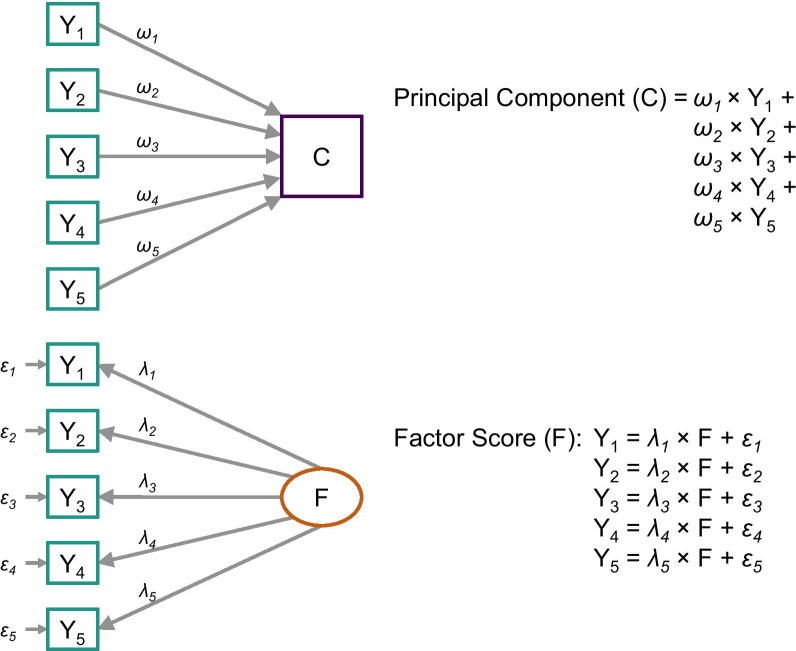
Development of the composite index of output. *Note* Principal component analysis and factor analysis was used to develop the composite index of output

To estimate health worker productivity as a measure of TE for multi-function HCs, we need to solve the complexity of these facilities producing multiple outputs. We used two different statistical methods—principal component analysis (PCA) and factor analysis (FA)—to estimate the summary measure of outputs (SMO) from the five output measures. Both PCA and FA are data reduction techniques that allow us to build a single measure from multiple variables capturing the most variability in the data, with some fundamental differences in the underlying theory and assumptions (Fig. 2).

As indicated in Fig. 2, using the PCA, we can develop a single index measure—also called a component (*C*)—which is the weighted average of indicators *Y*_1_ to *Y*_5_ [31]. From a causal perspective, it signifies that the five outputs are cumulatively producing the index measure that reflects the overall output of an HC. In contrast, FA considers there is a latent variable (*F*)—in this case, the overall or system-level outputs produced by an HC—which we cannot directly measure [32]. This latent construct represents itself through the common variance shared by some individual outputs, which we can measure. If *Y*_1_, *Y*_2_,*…Y*_5_ are highly correlated—indicating the same latent construct—we will see strong associations (*λ*_1_*, λ*_2_*,…λ*_5_) between the outputs and the latent variable (Fig. 2). The unique variance not explained by *F* is considered as the measurement error (*ε*_1_*, ε*_2_*,…ε*_5_). Parameterizing these equations, we can statistically estimate the factor score representing the latent construct’s value.

After estimating the PCA and factor score for each HC, we explored their consistency using the Pearson correlation coefficient and visualizing their distribution. As FA is theoretically suited for this analysis and produces a more precise measure, we used factor score as the SMO of each HC. We rescaled factor scores between 0 and 100 because the standardized factor scores generated from the FA presents a mean of 0 and a standard deviation of 1.

### Calculating the health worker productivity measure

We calculated the productivity of each HC by dividing the SMO by the total number of health staff, which is the cumulative number of HO equivalent clinical staff, para-clinical staff, and admin staff.

$${\text{Productivity}}\,{\text{score = }}\frac{{{\text{Summary}}\,{\text{measure }}\,{\text{of}}\,{\text{outputs (factor}}\,{\text{score }}\,{\text{rescaled}}\,{\text{from}}\,{ 0} - {100)}}}{{{\text{Total }}\,{\text{health}}\,{\text{workforce }}\,{\text{(HO}}\,{\text{equivalent}}\,{\text{ clinical}}\,{\text{staff }}\,{ + }\,{\text{ paraclinical }}\,{\text{staff }}\,{ + }\,{\text{ admin}}\,{\text{ staff)}}}}\,$$

The crude productivity score represents the average unit of the SMO per staff of the facility. We have also examined the relationship of the productivity score with the SMO and the staffing level of facilities.

### Developing examples of practical application of productivity score

We provided examples of how this kind of analysis could be used in practice by health system managers: (1) investigating the determinants of productivity as an explanatory tool for policymaking, and (2) ranking of the HCs and higher administrative levels using the adjusted productivity score. To develop these examples, we implemented multilevel linear mixed-effects regression models accounting for the confounding effect of the contextual factors. Contextual factors affect HCs’ capacity to produce outputs by influencing the service utilization volume, and subsequently, its productivity [25]. HCs may yield higher outputs when situated in an urban area due to higher demand. Likewise, a cluster of HCs located in a geographical area (woreda or zone) may have more health workers because of policy measures. The ranking of the HCs based on crude productivity can be misleading due to the confounding effect of the intrinsic and extrinsic contextual factors.

We accounted for the contextual factors and the clustering effects in the regression model to explore the determinants of productivity. We performed a log–log transformation of the dependent variable and the provider-mix covariates (number of HO equivalent clinical, para-clinical, and admin staffs) as they are highly skewed to the right [33]. The regression was used to estimate the predicted productivity of HCs, which is a more precise measure of productivity adjusted for the contextual factors. The descriptive analysis, PCA, and regression models were performed using Stata 15.1 [29], FA was performed using Mplus 8.3 [34], and visualizations of the results were developed using the R package ggplot2 3.3.3 [35].

## Results

### Descriptive exploration of the analytical dataset

**Fig. 3:**
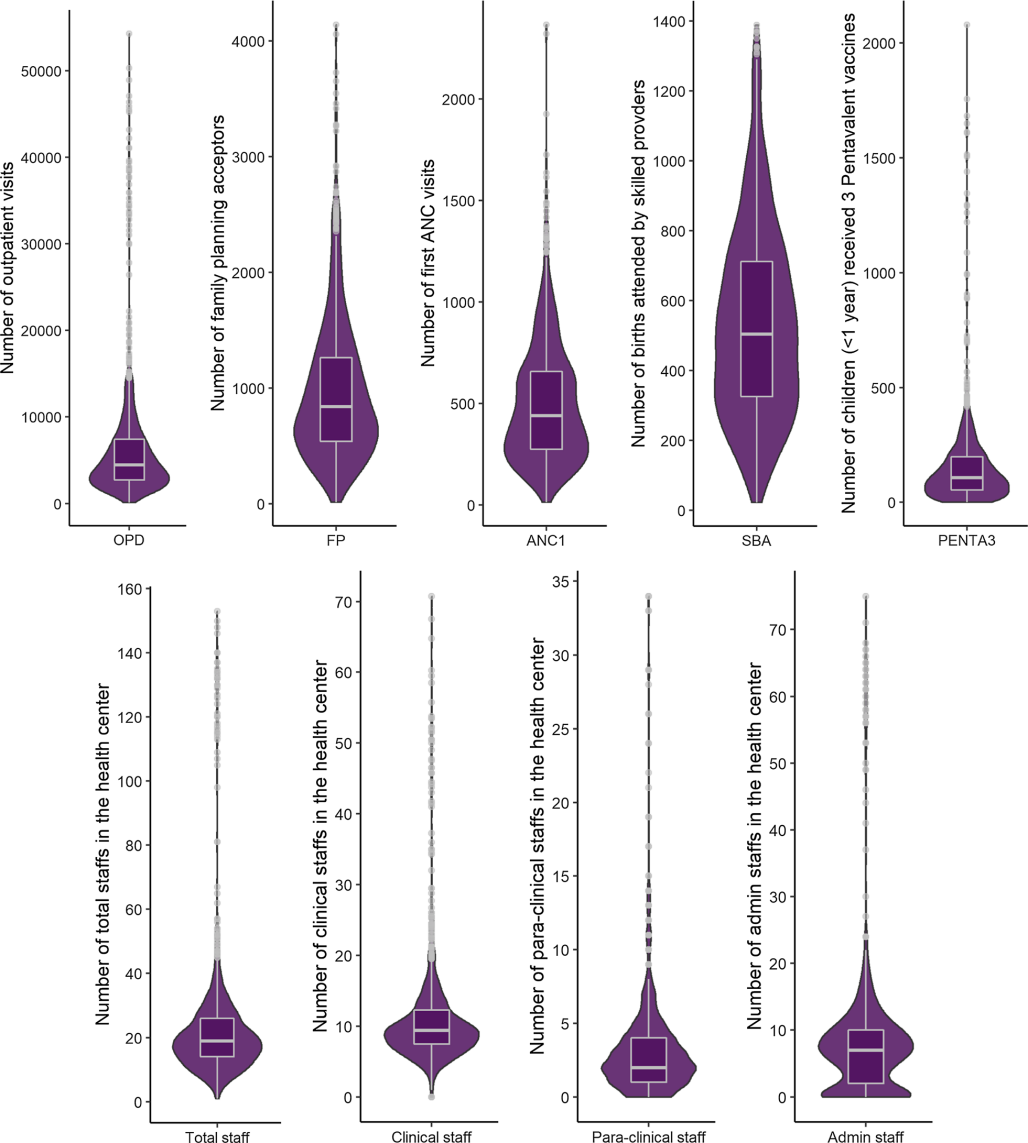
Distribution of the output and input measures from the health centers (*N* = 1128)

Our analytical sample included 1128 HCs from 369 woredas within 39 zones. We found substantial missingness and outliers for the output and input measures (Table 1). Significant variability was also observed across the HCs for all input and output measures (Fig. 3) (See Additional File 1).

**Table 1:** Output and input measures and their descriptive statistics

Indicators and their description		Facilities		Descriptive statistics	
		Total	Without outlier		
		*N* (%)	*N *(%)	Mean	SD
Output measures reported in the HMIS		*N* = 2163		*N* = 1128	
OPD	Total number of outpatients visits	2143 (99.08)	1729 (79.94)	6458.75	7063.92
FP	Total number of family planning acceptors	2136 (98.75)	1758 (81.28)	974.91	618.46
ANC1	Total number of pregnant women receiving their first antenatal care visit	2143 (99.08)	1815 (83.91)	500.97	299.34
SBA	Total number of births attended by a skilled provider	2137 (98.80)	1822 (84.23)	536.18	273.85
PENTA3	Total number of children (less than 1 year) received three pentavalent vaccines	1905 (88.07)	1731 (80.02)	158.00	202.53
Input measures reported in the HRIS		*N* = 2005		(*N* = 1128)	
Health officers equivalent clinical staff^a^	Total number of doctors, emergency surgical officers, health officers, nurses, and midwives adjusted for their years of training	1645 (82.04)	1579 (78.75)	11.30	8.41
Para-clinical staff	Total number of lab technicians, pharmacy technicians, environmental technicians, anesthetists, other health professionals	1508 (75.21)	1392 (69.43)	3.51	3.92
Admin staff	Total number of administrative staff	1362 (67.93)	1341 (66.88)	8.59	10.59
Total	Sum of health officer equivalent clinical staff, para-clinical staff, and admin staffs	1645 (82.04)	1593 (79.45)	23.71	21.55

^a ^While health officers and nurses receive four years of training, doctors and emergency surgical officers receive six years of training, and midwives receive three years of training. Considering the health officers’ years of training as the reference, we have calculated a weighted value of the total clinical staff

After removing the outliers, we have identified 1582 health centers that reported all output measures, and 1483 health centers reported all input measures; The merged dataset of the input and output measures contains 1128 health centers from 369 woredas within 39 zones of Addis Ababa, Oromiya, and SNNPR

### Estimation of the summary measure of outputs and its descriptive exploration

**Fig. 4:**
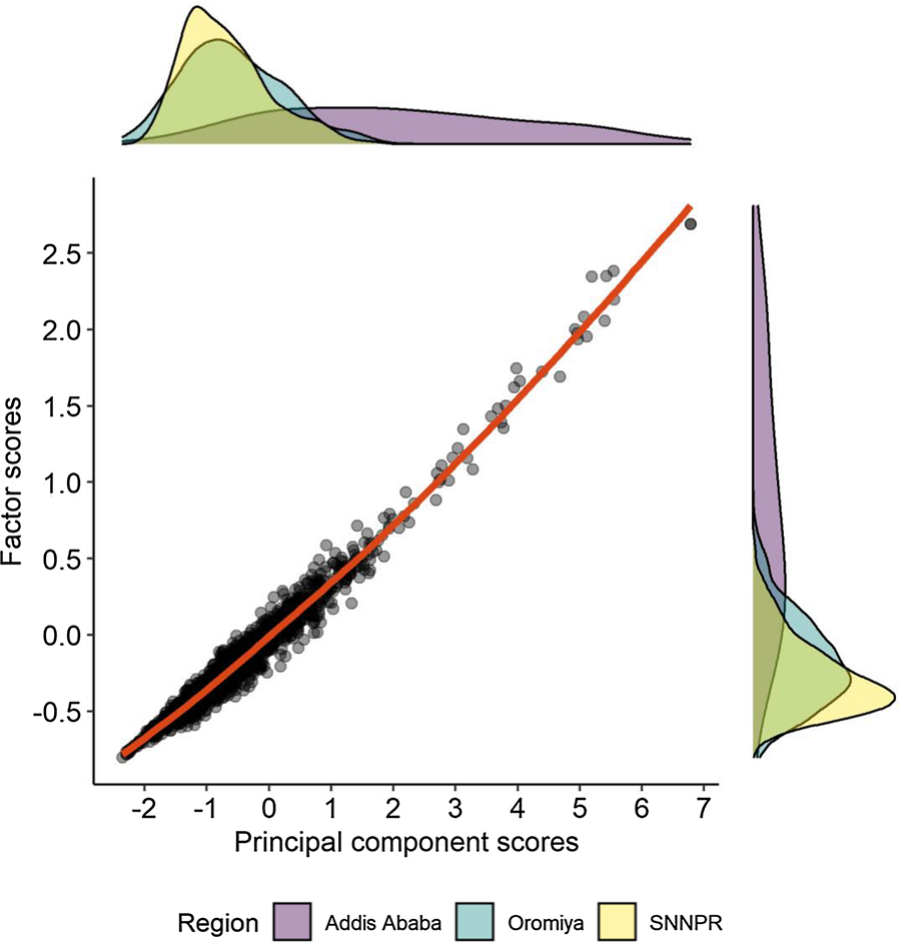
Consistency between the summary measure of outputs—PCA and FA measures. *Note* The red line indicates the locally weighted smoothing (lowess) curve

The first component extracted from the PCA explained ~ 63% of the total variance presented by the five output measures. Before performing FA, we assessed the possible number of latent constructs that could emerge from the data using Horn’s parallel analysis [36], which indicated that only one latent measure could be extracted from the data, consistent with our conceptualization. We implemented the factor analytical model with one latent measure, which presented adequate goodness of fit to the data [37]. We observed robust factor loadings and relatively smaller residuals, indicating a high construct validity of the observed latent measure [38] (See Additional File 1). The Pearson correlation coefficient between PCA and factor scores was 0.98 (*p* < 0.001), suggesting a very high degree of consistency (Fig. 4). Moving forward, we have used the factor score as the SMO.

**Fig. 5:**
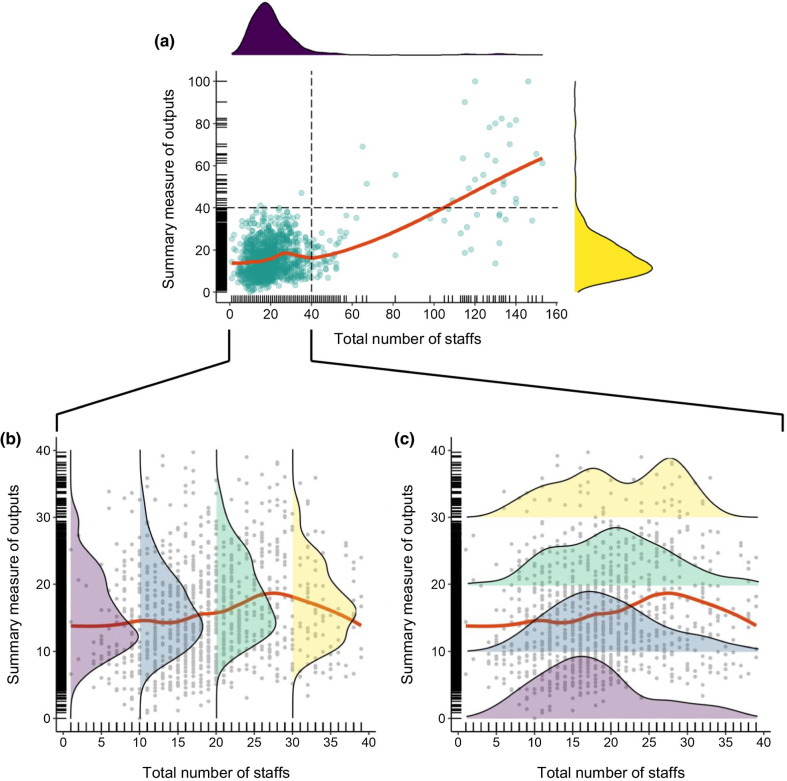
Relationship of total staff and summary measure of outputs of the health centers. *Note* **(b)** and **(c)** examine the staff and output relationship at lower staffing levels in more detail. For the ease of visualization, the range of total staff and output was truncated from 0 to 40. The red line indicates the locally weighted smoothing (lowess) curve. The density curves in **(b)** and **(c)** represent the observations between the sections of 0–10, 11–20, 21–30, and 31–40

We explored the relationship between the SMO and the total staff to understand the variability of the outputs across the level of the health workforce. A strong relationship between staffing and output would be represented by a consistent diagonal line. Instead, we find a little relationship at the typical level of HC staffing and some increase in output with substantial increases in staffing. This is a weak, but positive, association between the production output and the total number of staff (Fig. 5a).

More than 97% (*n* = 1100) of HCs had an SMO between 0 to 40, and within that range, we observe considerable variability. For example, with the same staffing level (0–10), the SMO of the HCs was substantially different, indicated by the vertical dispersion (Fig. 5b). A similar horizontal dispersion was observed in the data. For instance, the SMO of 11 to 20 was observed for HCs with a wide range of staffing (Fig. 5c). The almost horizontal red line representing the average relationship disguises the large variability at each staffing level, which is a far more critical observation from a TE perspective. Reducing this variation by improving productivity in facilities with low output relative to the staff at each level would be a practical management objective for improving productivity.

### Productivity ratio and its descriptive exploration

A low productivity score was observed for the HCs included in the study with a mean of 0.98 (*N* = 1128, range 0.00–6.59). The distribution of productivity scores of HCs was substantially different from their SMO. While the SMO was highest in Addis Ababa (*N* = 41, mean = 51.02), followed by Oromiya (*N* = 717, mean = 16.94) and SNNPR (*N* = 369, mean = 13.69), the highest health workforce productivity was observed for Oromiya (*N* = 717, mean = 1.19), followed by SNNPR (*N* = 369, mean = 0.64) and Addis Ababa (*N* = 41, mean = 0.43).

**Fig. 6:**
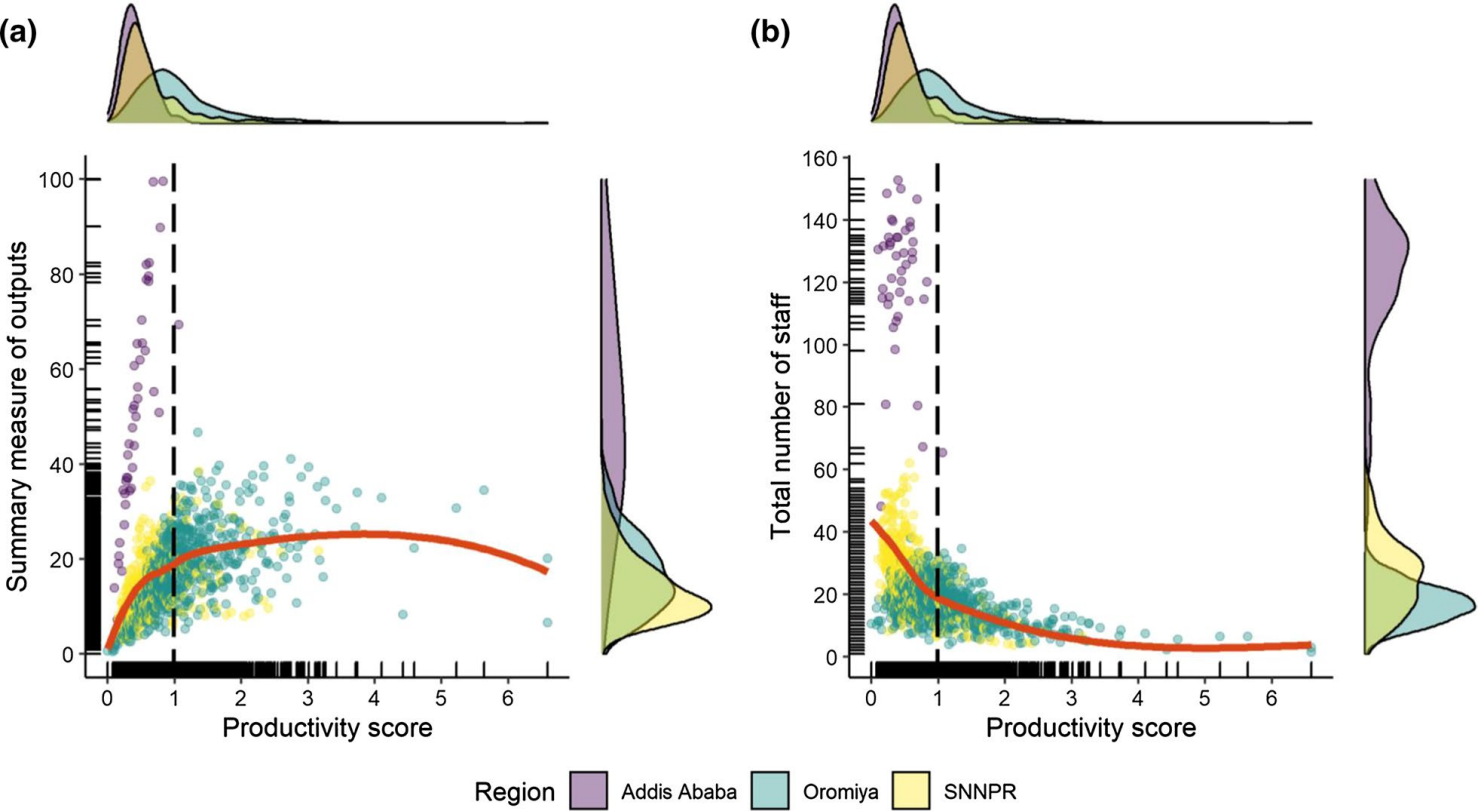
Relationship of the productivity with the summary measure of outputs and total staffs. *Note* Red lines indicate the locally weighted smoothing (lowess) curve for the total sample, the black dashed line represents the average productivity score of the health centers (0.98)

The association between the SMO of an HC and the productivity presented an inverted U-shape relationship (Fig. 6a: red line). In the initial stage (black dashed line: average productivity score of 0.99), we observe a proportionate increase in SMO with an increase in productivity of the health workers. Next, for Oromiya and SNNPR, the SMO plateaued, and any further increase in productivity of health workers did not increase the SMO. However, in Addis Ababa, productivity remained low regardless of the output level, indicating a significant level of insufficiency of the HCs.

The relationship between productivity and staffing level is presented as an upward concave curve (Fig. 6b: red line). With more staff, the productivity of the health workers decreased and vice versa. HCs in Addis Ababa had lower productivity than the other regions, which may reflect the overstaffing of health facilities in Addis Ababa relative to the demand. Whereas, with fewer staff, HCs from Oromiya and SNNPR showed a wide variation of productivity. Ideally, if staff were equally productive and perfectly allocated to where they are needed, we would expect a vertical line, such as the black dashed line displaying an average productivity score of 0.99.

### Examples of practical applications of the productivity ratio

Table 2 presents the unadjusted and adjusted estimates from the regression models. Intraclass correlation coefficient (ICC) estimated by the null model suggested 51% and 28% of the total variation of productivity was attributed to woreda and zone-level variation, respectively. In the final multiple regression (Model 3), the productivity of HCs presented a negative association with the number of clinical and admin staff (*p* < 0.001). Accounting for all the fixed and random effects, a 1% increase in the clinical and admin staff was associated with a 0.25% and 0.22% decrease in productivity. In contrast, both the number of beds in the HC and the woreda population presented a statistically significant positive association with the productivity score. In the adjusted model, no significant difference in productivity was observed between Addis Ababa and Oromiya. Comparing with Oromiya, the effect estimate for SNNPR attenuated from 0.79 in the unadjusted model to 0.85 (*p* < 0.001) in the adjusted model.

**Table 2:** Determinants of the productivity estimated by multilevel linear mixed-effects models

Covariates	Unadjusted model	Null model	Model 1	Model 2	Model 3
	*Est*	*Est*	*Est*	*Est*	*Est*
Fix effects					
Intrinsic contextual covariate: provider-mix					
HO equivalent clinical staff^a^	− 0.44^***^		− 0.25^***^	− 0.26^***^	− 0.25^***^
Para-clinical staff^a^	− 0.16^***^		− 0.04	− 0.04	− 0.04
Admin staff^a^	− 0.26^***^		− 0.23^***^	− 0.22^***^	− 0.22^***^
Intrinsic contextual covariate: facility size					
Number of beds in health centers^b^	1.003			1.004	1.005^**^
Extrinsic contextual covariate					
Woreda population (per 10,000)^b^	1.004^**^				1.004^***^
Region (Ref: Oromiya)^b^					
Addis Ababa	0.67^***^				0.98
SNNPR	0.79^***^				0.85^***^
Constant		0.85^***^	1.32^***^	1.29^***^	1.28^***^
Random effects					
Zone-level residual variance		0.02	0.02	0.02	0.008
Zone-level ICC		0.28	0.25	0.25	0.16
Woreda-level variance		0.02	0.01	0.01	0.01
Woreda-level ICC		0.51	0.44	0.44	0.36
Observations	1128	1128	1128	1128	1128
AIC		*55.78*	*− 232.26*	*− 233.63*	*− 255.90*
Log-likelihood		*− 23.89*	*123.13*	*124.81*	*138.95*

The analytical sample is consisting of 1128 health centers, from 369 nested within 39 zones

The dependent variable was log transformed to achieve the normality as it was highly skewed to the right

*ICC* intraclass correlation; *Est* effect estimates

^a^Variables were log transformed to achieve the normality as they are highly skewed to the right. Their associated effect estimates present the elasticity of provider-mix

^b^Effect estimates of the variables were exponentiated for ease of interpretation

***p < 0.001, **p < 0.01, *p < 0.05

**Fig. 7:**
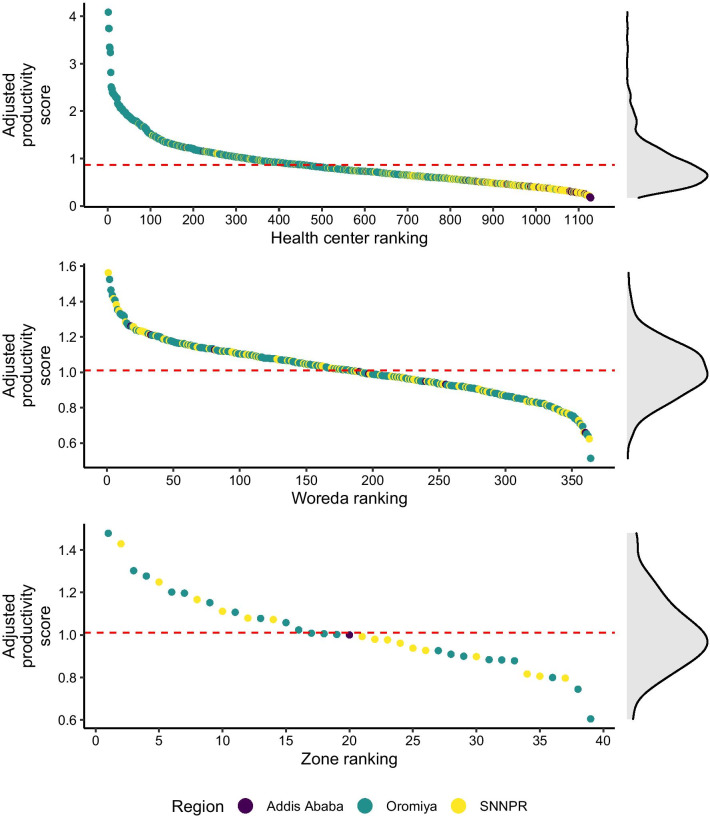
Ranking of health centers, woredas, and zones based on health centers productivity. *Note* The red line indicates the mean adjusted productivity of the health workforce at the level of health centers (0.86), woredas (1.01), and zones (1.01)

The regression models also allowed us to estimate the adjusted health workforce productivity, which was used to rank HCs, woredas, and zones according to their adjusted average productivity, accounting for the clustering effect and contextual factors. Figure 7 presents the ranking of HCs, woredas, and zones based on the predicted productivity scores. Though the HCs from Addis Ababa ranked the lowest in productivity based on woreda and zone-level productivity Addis Ababa’s ranking moved upwards. Like the crude productivity score, the adjusted productivity of HCs was skewed to the right. However, the adjusted productivity of the woreda and zone was much more normally distributed.

## Discussion

This study demonstrates an innovative analytical approach using routine health facility data to estimate a summary measure of facility outputs and the health workforce’s productivity as TE measures for PHC facilities. We developed the SMO for each HC from five output measures using FA. Next, crude productivity scores were formed using the ratio of SMO and the total staff number. Regression models enabled a second analytical step to estimate the adjusted productivity scores accounting for several contextual covariates. Lastly, we presented two motivating examples of the use of the productivity scores.

Ratio-based analyses are typically limited to one input and one output [25]. Using the FA to estimate a summary measure of multiple outputs and aggregating the total number of health workers (inputs) demonstrates a practical approach for addressing the “multiple-input multiple-output” problem in analyzing TE using large-scale routinely available data. While we believe the five outputs included in FA reflect the majority of the services produced by HCs, it is possible to expand the list by including indicators related to other outpatient services (e.g., tuberculosis, malaria, HIV), inpatient care, nutritional services. Summary measures of output can also be linked to the cost of facilities (e.g., operational cost or cost of services) [25].

Descriptive analyses highlighted the large variability in productivity across similar health facilities in Ethiopia, represented by the significant dispersion in SMO occurring at similar levels of the workforce (Fig. 5b and c). Many productivity studies only focused on the average relationship between output and staffing represented by the regression line [39–41]. This grossly misrepresents the large variability in productivity, which should be the target of efforts to improve TE. A technically efficient health system will not only show a strong positive association between the outputs and level of staff (a steeper regression line in Fig. 5b and c) but also exhibit lower dispersion across the regression line.

Association of SMO and staffing provides a simple measure of TE and offers guidance to policymakers and managers about improving productivity. A number of contextual factors likely confound the health workforce productivity (such as population density, demand, and access to care, urbanization, etc.). We provide an example to account for the effects of such covariates using a multilevel linear mixed-effects model [42]. While implementing DEA analysis, other studies have also taken a similar approach to account for the contextual factors [43–45]. After accounting for confounders, 36% and 16% of the variability of productivity was attributed to woreda and zone, respectively, indicating a significant contribution of the geographical location on TE. Better data may be useful to analyze what demand and supply factors are represented by this geographic association. This is critical because policy and management processes are designed and implemented, not considering each health facility’s characteristics, rather than at the administrative level [21]. Ranking HCs, woredas, and zones according to the productivity of HCs could provide guidance on where to target interventions to improve TE.

This study demonstrates the feasibility and usefulness of this approach. Due to the use of older data and problems with data quality (missingness and outliers), the results presented here are not applicable for HCs in Ethiopia today. We are working with Ethiopia’s Ministry of Health to apply this approach to more recent and better quality data now being produced in Ethiopia’s DHIS2 system [46]. We are also carrying out a qualitative study on productivity to explore explanatory factors that could support interventions to improve productivity and TE. Understanding the causes of low productivity and developing approaches for improvement is the intended outcome of work of this kind.

## Conclusions

Improving the TE of PHC delivery in LMICs is essential to improve health system performance. A richer understanding of the production processes in health facilities in LMICs is still quite limited [47], yet critical to design effective policy and practice to improve TE. While various approaches can advance this important work, one useful contribution would be to create a simple enough measure that can be calculated at the facility or subnational levels using routinely available data. The use of simple metrics like productivity ratio has a practical use for health systems management purposes, in contrast to complex analytical approaches like DEA or SFA, which are often difficult to grasp by the policymakers and health managers [6]. The methodology presented in this study can be readily applied in LMICs using widely available data that will allow further explorations to understand the causes of inefficiencies in the health system. The strategic direction provided by such analysis will help managers and policymakers to undertake actionable measures and monitor the progress of universal health coverage.

## Supplementary Information

**Additional file 1.** Categorization of healthcare workers. Distribution of the output and input measures with outliers. Result of Horn’s parallel analysis and factor analysis (N = 1128).

## Data Availability

The data used in this analysis may be requested to Ethiopia’s Ministry of Health by any qualified researchers.

## Abbreviations

AE: Allocative efficiency
AIC: Akaike information criterion
ANC1: First antenatal care visits
DEA: Data envelopment analysis
DHIS2: District Health Information Software-2
DRGs: Diagnostic-related groups
FA: Factor analysis
FP: Acceptors of modern family planning methods
HC: Health Center
HMIS: Health management information systems
HRIS: Human resource information system
ICC: Intraclass correlation coefficient
LMIC: Low- and middle-income country
OPD: Outpatient visits
PCA: Principal component analysis
PENTA3: Children with three pentavalent vaccines received within their first year
PHC: Primary healthcare
SBA: Facility-based deliveries by skilled birth attendants
SFA: Stochastic frontier analysis
SMO: Summary measure of outputs
SNNPR: Southern Nations, Nationalities and People’s Region
TE: Technical efficiency
