# Supplementary material for: A practical measure of health facility efficiency: an innovation in the application of routine health information to determine health worker productivity in Ethiopia
Source: Hum Resour Health. 2021 Aug 5;19:96. doi: 10.1186/s12960-021-00636-6 (PMC8340541; doi:10.1186/s12960-021-00636-6)
Supplement: Supplementary file 1 — Additional file 1. Categorization of healthcare workers. Distribution of the output and input measures with outliers. Result of Horn’s parallel analysis and factor analysis (N = 1128). [file 12960_2021_636_MOESM1_ESM.docx]

A practical measure of health facility efficiency: An innovation in the application of routine health information to determine health worker productivity in Ethiopia

***Additional File 1: Supplemental Materials***

**Supplementary Material 1:** **Categorization of healthcare workers**

| **Variable Name** | **Type of healthcare workers** |
| --- | --- |
| Clinical staffs | Male and female doctors |
|  | Male and female emergency surgical officers |
|  | Male and female health officers |
|  | Male and female nurses |
|  | Male and female midwives |
| Paraclinical staffs | Male and female laboratory technicians/technologists |
|  | Male and female pharmacy technician/pharmacist |
|  | Male and female environmental technician/environmentalist |
|  | Male and female anesthetists |
|  | Male and female other health professionals |
| Admin staffs | Male and female administrative personnel |

**
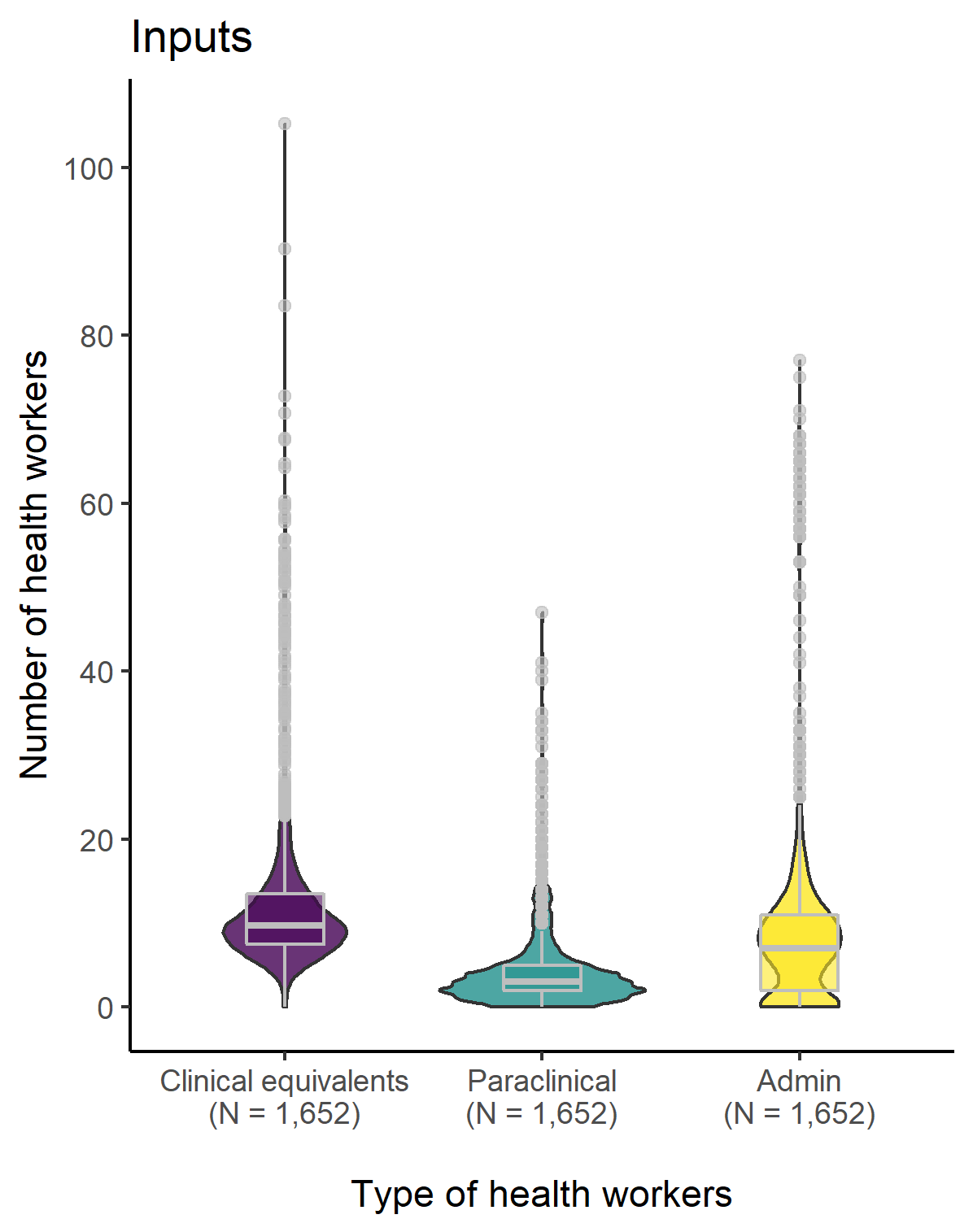
Supplementary Material 2:** **Distribution of the output and input measures with outliers**


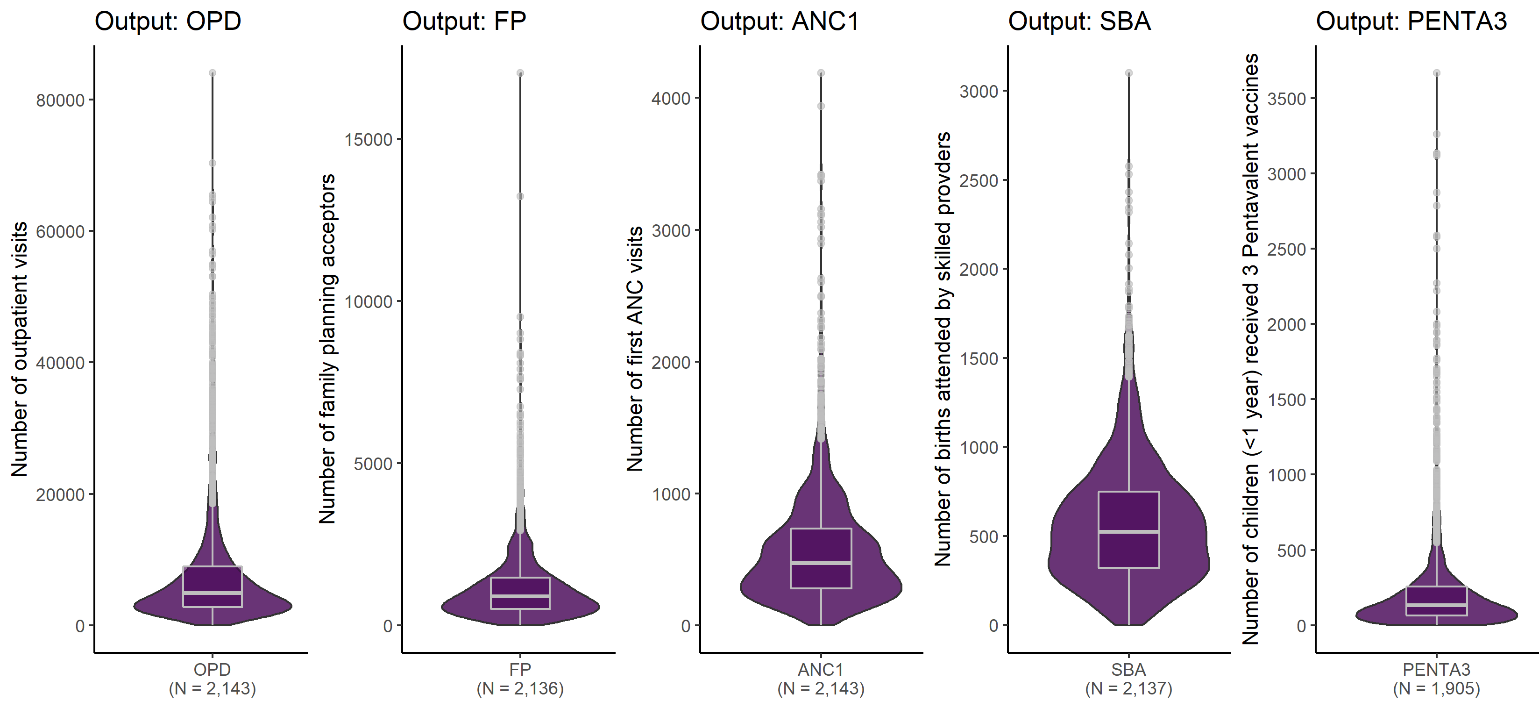


**
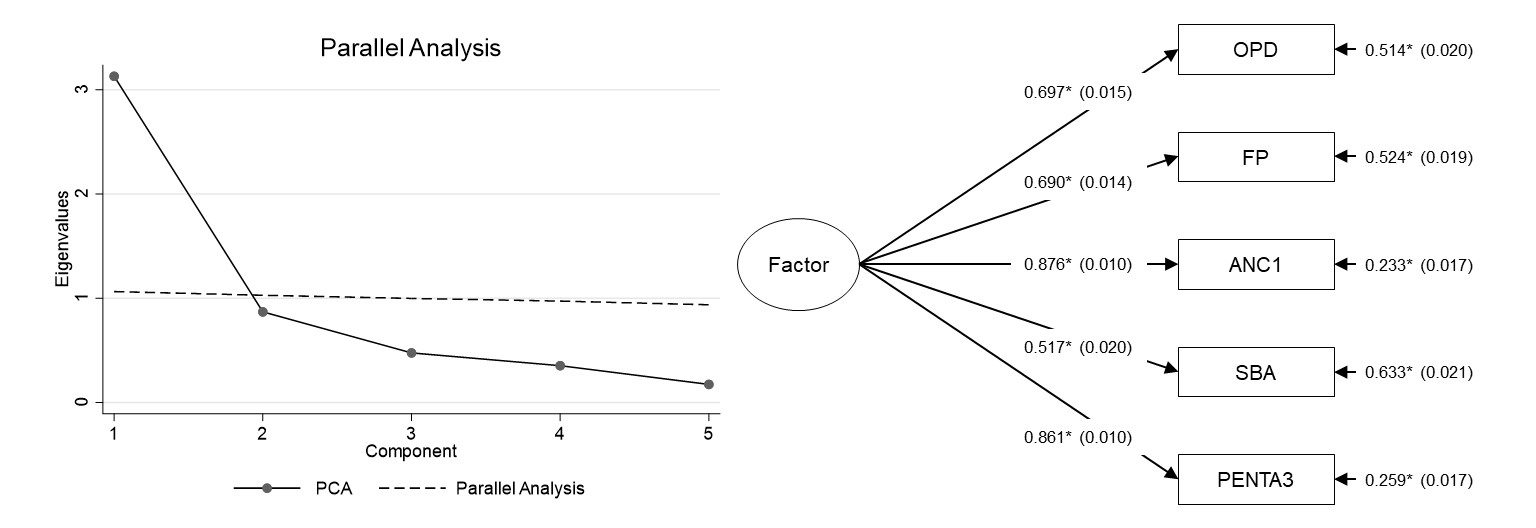
Supplementary Material 2:** **Result of Horn’s parallel analysis and factor analysis (N = 1,128).**

Note: * = P < 0.001; The factor analysis is implemented using the Maximum Likelihood (ML) estimation; Goodness of fit indices: root mean square error of approximation (RMSEA) = 0.073, comparative fit index (CFI) = 0.920, Tucker-Lewis index (TLI) = 0.890, standardized root mean square residual (SRMR) = 0.056, λ^2^ value = 652.105, df = 5, p < 0.001.
